# Pollinator limitation on reproductive success in *Iris tuberosa*

**DOI:** 10.1093/aobpla/plu089

**Published:** 2014-12-19

**Authors:** Giuseppe Pellegrino

**Affiliations:** Department of Biology, Ecology and Earth Science, University of Calabria, I-87036 Rende, Italy

**Keywords:** Fruit set, hymenopteran insects, Iridaceae, pollinators.

## Abstract

Variation in plant and floral size can have conflicting effects on pollination and fruit production in flowering plants. This research examines the contributions of plant height, flower size and pollinator visitation to reproductive success in four populations of *Iris tuberosa*. The plants were pollinated exclusively by hymenopteran species, primarily during sunny days. Pollination supplementation increased the proportion of flowers that matured into fruit, with 95 % fruit set for hand-pollinated compared with 74.15 % for naturally pollinated flowers. The pollinator visitation rate and the proportion of fruit produced were not significantly different between tall and short plants or between small and large flowers. Furthermore, the increase in plant size and floral display did not increase the frequency of pollinator visitations and so did not increase the fruit set. Thus, despite the widespread effects of flowering plant size on pollinator attraction and plant reproduction in other species, these effects are lacking in *I. tuberosa*. This study quantifies the role of pollinators in the reproductive success of *I. tuberosa*. Pollinators visited tall/short plants and large/small flowers in equal proportion, suggesting that plant and floral display size do not affect pollinator attraction and reproductive success in *I. tuberosa*. These results suggest that sexual reproduction of *I. tuberosa* is fairly limited by pollinators and not by resource limitation.

## Abstract (precis)

In this paper I measured the effect of varying pollinator visits on fruit production in order to understand pollination strategy in *Iris tuberosa*, and assessed the effects of plant and floral display size on pollination to understand how these factors influence reproductive success. This study found that *I. tuberosa* were pollinated exclusively by hymenopteran species, mainly during sunny days, and that plant and floral size did not affect fruit set and pollinator attraction. Thus, the sexual reproduction of *I. tuberosa* is fairly limited by pollinators and not by resource limitation.

## Introduction

Plants should invest in flower production until visitation yields the amount of pollen necessary to produce the maximum number of seeds that resources can support (Haig and Westoby 1988), and thus plants would be simultaneously limited by resources and by pollen. The frequency and potential consequences of pollen limitation for plant populations and communities have been intensely explored during the last few decades (Ashman *et al.* 2004; García-Camacho and Totland 2009; Gómez *et al.* 2010; Harder and Aizen 2010). Pollinator limitation of female reproduction occurs when an inadequate supply of pollen limits fruit set below the level possible given the plant's available resources. A decrease in pollinator frequency (i.e. number of visits) is likely to decrease the quantity of pollen deposited onto the stigma (Ashman *et al.* 2004), reducing fruit and seed set (Bierzychedek 1981; Matsui *et al.* 2001; Pellegrino *et al.* 2005; Aizen and Harder 2007). Moreover, the plant's ability to attract the pollinators by means of flowers can be a crucial component of fitness. Indeed, studies have generally found a significant relationship between the response of pollinators and increasing plant and flower size (Conner and Rush 1996; Ohashi and Yahara 1998; Ashman *et al.* 2000; Glaettli *et al.* 2008). In fact, limitation of fruit and seed production by insufficient pollinator visitation is common and ubiquitous across angiosperms (Ashman *et al.* 2004; Knight *et al.* 2005).

Although the family Iridaceae is a well-defined assemblage of ∼1800 species belonging to the Liliidae, little information is available on the role of pollinators in their reproductive success. The derived features of Iridaceae include two morphological characters, a unifacial isobilateral leaf and the presence of only three stamens (Goldblatt 1990). Except for the Tasmanian genus *Isophysis*, the flowers of Iridaceae also have an inferior ovary, and both septal and perigonal nectaries (Goldblatt 1990). The majority of species of Iridaceae are pollinated by Hymenoptera (mostly bees). It is now evident that pollination systems are predominantly specialist; plants rely on a single species or a few ecologically analogous species for pollination (Goldblatt and Manning 2006). Attractants are primarily perianth pigmentation, complemented by a range of floral odours in many species, but flower shape and tepal orientation, in particular functional floral symmetry, may be equally important for some pollinators.

*Iris*, the largest genus of the Iridaceae with ∼250 species (Mathew 1981), extends across the Earth's North Temperate Zone to North America, and in Europe and Asia (Khassanov and Rakhimova 2012). Early studies on pollination in *Iris* showed that the species were visited by bumblebees (Segal *et al.* 2006; Imbert *et al.* 2014). Each outer tepal and its opposed petaloid style crests function as a bilabiate pollination unit, and thus to a bee appear as a single gullet flower. A pollinator visiting the flower in search of nectar alights on the lower lip (outer tepal limb) and pushes its head and mouthparts into the gullet (the space between the tepal claw and style branch). In doing so its upper thorax passively brushes against the anther, becoming dusted with pollen. Visiting another flower, its thorax will brush against the stigmatic lobe transferring any pollen onto the receptive tissue. *Iris tuberosa* (subfamily Iridoideae) has received very little attention. It has a characteristic floral morphology: every flower has three units, each containing one stamen and a stigma enclosed by a petaloid style and a sepal. To date, no information is available on the pollination strategy of *I. tuberosa*; however, the plant needs an appropriate pollinator visiting the flower to gather the nectar located at the base of tepals, as observed by Arcangeli (1895), who identified the hymenopterous *Xylocopa violacea* as a pollinator of *Hermodactylus tuberosus* (synonym of *I. tuberosa*). In *I. tuberosa*, self-pollination is very difficult due to the location of the anthers under stigma lobes.

In this paper, I measured the effect of varying pollinator visits on fruit production in order to understand the pollination strategy in *I. tuberosa*, and assessed the effects of plant and floral display size on pollination to understand how these factors influence reproductive success. The aims of this research were to: (i) determine the pollinators and the fruit production under natural conditions, (ii) evaluate the influence of pollinators on reproductive success, (iii) test if plant and floral size affect pollinator attraction and fruit set and (iv) define the breeding system of *I. tuberosa*.

## Methods

### Study site and species

**Figure 1.**
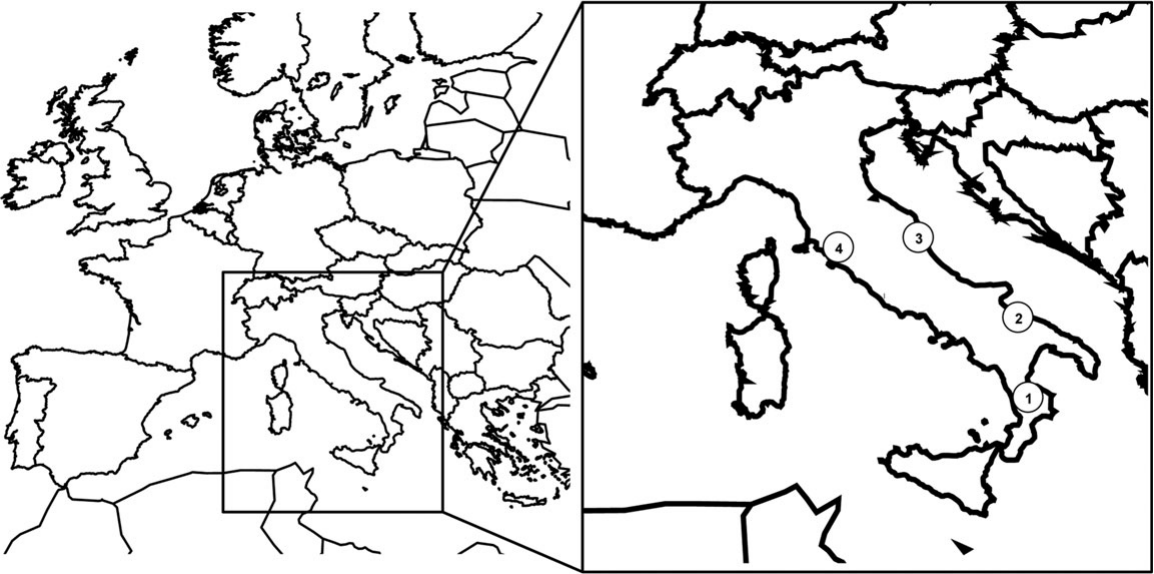
Location of four populations: Cassano (1), Corato (2), Acquarola (3) and Lucignano (4) of *I. tuberosa* in central-southern Italy.

*Iris tuberosa* is a native species of Mediterranean regions including Southern Europe, the Balkans and Northern Africa (Mathew 1987). In Italy, where I conducted fieldwork, the species occurs mainly in the country's central and southern regions where it grows in dry, usually rocky places, in olive groves, and among hedges (Pignatti 1982). Flowers of *I. tuberosa* are hermaphroditic and trimerous, thus consisting of two whorls of petal-like members (a brownish outer and a greenish inner series of tepals), with three stamens inserted opposite to the outer tepals and an inferior ovary of three united carpels sharing a common style. Information regarding its reproduction system is rather scarce. Although *I. tuberosa* ovaries contain over 100 ovules, each produces capsules with only a few mature seeds (9–12 seeds), which are randomly distributed along the placentas, due to the low germination rate and the low number of pollen tubes which reach the ovary (Grilli Caiola and Brandizzi 1997; Grilli Caiola *et al.* 2000). Observations were conducted during the flowering period of *I. tuberosa* in March–May of 2012 and 2013 in four sites called Cassano, Corato, Acquarola and Lucignano in central-southern Italy (Fig. 1). To minimize the effects of soil and vegetation types on measurements, I chose sites of matched vegetation types. All sites consist of calcareous, dry grasslands (Festuco-Brometalia); *Spartium junceum* L., *Cytisus sessilifolius* L. and *Cistus incanus* L. are the frequent shrubs and *Festuca circummediterranea* Patzke, *Bromus erectus* Huds. and *Dactylis glomerata* L. are the dominant herbs.

### Plant and floral size

To test the effects of plant size on pollinator attraction and fruit set, at the peak of the blooming season (March–April) I measured phenotypic traits of 20 plants in each site. Plant height (the distance from the ground to the top of the flower) was measured using a ruler. Flowers were photographed under a binocular microscope with a digital camera. The length and width of the outer brownish tepals (platform for pollinators) were measured from these images using the program Analysis (Soft Imaging Systems, Münster, Germany). For each population, plants were divided into two groups as ‘tall’ and ‘short’ plants, which were taller or shorter than the mean height, respectively.

### Flower-visiting insects

Insect visitation was recorded during the peak flowering season, from 2 March to 16 April 2012 and from 6 March to 21 April 2013, for a total of 80 h for sites in each year. Sampling of flower-visiting insects was restricted to 7–8 days at each location in order to minimize any negative impact on the local insect fauna and to minimize negative effects on the fruit set. The sites were intermittently visited during the daytime (daily between 0800 and 1800 h). Pollinators found on *I. tuberosa* flowers were captured with a hand net and checked for pollen. A visitor was considered a pollinator if the insect touched either the anther or the stigma of the flower and had *Iris* pollen on its body. The insects were then put down with ethyl acetate and stored individually for later identification. The species for each specimen was then identified using the taxonomic keys from Schmid-Egger and Scheuchl (1997), and the voucher specimens deposited in the Laboratory of Plant Biosystems of DiBEST of University of Calabria.

### Fruit set and breeding system

As unmanipulated open-pollinator controls, 15–20 smaller than average plants and the same number of larger plants were left unbagged to pollinators in each site, each year. At the end of the flowering period (April–May), the flowers that produced fruits were recorded and reproductive success was calculated as the ratio between the number of fruits produced and the number of available flowers.

Hand-pollination treatments to determinate the breeding system of *I. tuberosa* were performed from 20 March to 10 April 2012 and from 16 March to 10 April 2013. In each population, ∼30 plants were bagged with a fine-meshed cloth to exclude pollinators. For hand pollination, the cover was removed, flowers were pollinated, marked with cotton thread and the cover replaced. Each flower was randomly assigned to one of four hand-pollination treatments: (i) covered but without manipulation to test for spontaneous autogamy, (ii) emasculated to test for agamospermy, (iii) artificially self-pollinated and (iv) outcrossed or by induced xenogamy. For self-pollination, the pollen was transferred using a cotton stick from the same flower (induced autogamy). For outcrossing, pollen was transferred with a cotton stick from a flower to the stigma of another flower, previously emasculated and located at a distance of at least 10 m. In May, the number of capsules was counted and the ratio between the number of flowers treated/fruit produced was determined for each hand-pollination treatment.

### Data analysis

Standard descriptive statistics and one-way analysis of variance (ANOVA) were performed for each quantitative parameter for morphological data. Morphological data were normally distributed and thus were not transformed. When the *F* test was significant, means were compared using the Tukey test at 5 % error probability. The effects of plant and floral size on measures of reproductive success (number of produced fruits) and the attraction of pollinators were compared with a *χ*^2^ test (Siegel 1979). The statistical program package SPSS (version 10, SPSS, Inc., Chicago, IL, USA) was used. I calculated self-compatibility indices (SCI) following Lloyd and Schoen (1992): SCI = fruit set after hand selfing/fruit set after hand outcrossing.

Self-compatibility index ranges from 0 to 1, with 1 representing full self-compatibility.

A population of plant was considered self-compatible if SCI exceeded 0.75 and if at the same time no statistically significant difference was detected between seed set after hand selfing and hand outcrossing. I calculated a pollen limitation index (PLI) where

$$\begin{array}{r} \text{PLI = }(\text{fruit}\;\text{set}\;\text{hand}\;\text{outcrossing}-\text{fruit}\;\text{set}\\ \text{open}\;\text{pollination})/\mathrm{fruit}\;\text{set}\;\text{hand}\;\text{outcrossing} \end{array}$$

This index compares the difference between the fruit set following supplementary hand pollination and the fruit set following natural pollination, divided by the former, and represents the potential for fruit production when pollination is not limited. Pollen limitation index values above zero indicate pollen limitation.

## Results

### Plant and floral size

Plant height of *I. tuberosa* ranged from 18.00 to 39.50 cm (mean value 27.43 ± 1.92 cm) (Table 1), and there were no significant differences between the populations with regard to average plant length (*F*_3,77_ = 0.97, *P* = 0.28 by ANOVA). In relation to the structure of floral traits, the outer tepal size of examined flowers ranged from 11.35 to 13.11 mm in width and from 18.85 to 21.34 mm in length (mean value 12.11 ± 0.95; 19.57 ± 2.02 mm) (Table 1). There were no significant differences between the populations regarding outer tepal size (width: *F*_3,37_ = 0.89, *P* = 0.43; length: *F*_3,37_ = 0.92, *P* = 0.31 by ANOVA). The flower and plant size were relatively constant among plants and among populations. All examined individuals produced an outer brownish tepal without significant differences in floral size.

**Table 1.** Summary of plant and floral size, and comparison of fruit set between ‘tall’ plants (taller than local mean height) and ‘short’ plants (shorter than local mean height) of four *I. tuberosa* populations. Data are presented as means ± standard deviation.

	Plant height (cm)	Outer brownish tepal		Fruit set (%)		
		Length (mm)	Width (mm)	Total	Tall plants	Short plants
Cassano (*n* = 20)	27.70 ± 2.15	19.65 ± 2.10	12.91 ± 0.95	72.10 ± 2.50	70.95 ± 1.75	73.25 ± 2.30
Corato (*n* = 20)	26.90 ± 1.90	18.96 ± 1.85	12.54 ± 0.93	75.25 ± 2.80	76.15 ± 2.10	74.35 ± 1.90
Acquarola (*n* = 20)	27.25 ± 1.75	19.98 ± 2.21	12.87 ± 0.92	80.35 ± 3.10	82.10 ± 2.40	78.60 ± 1.50
Lucignano (*n* = 20)	27.90 ± 1.90	19.71 ± 1.95	12.82 ± 0.98	68.85 ± 2.10	67.35 ± 1.50	70.35 ± 1.85
Mean	27.43 ± 1.92	19.57 ± 2.02	12.11 ± 0.95	73.75 ± 2.60	74.15 ± 2.00	74.15 ± 1.90
ANOVA	*F*_3,77_ = 0.97, *P* = 0.28	*F*_3,37_ = 0.92, *P* = 0.31	*F*_3,37_ = 0.89, *P* = 0.43	*F*_3,37_ = 0.69, *P* = 0.33	*F*_3,37_ = 0.72, *P* = 0.31	*F*_3,37_ = 0.59, *P* = 0.23

### Flower-visiting insects

**Figure 2.**
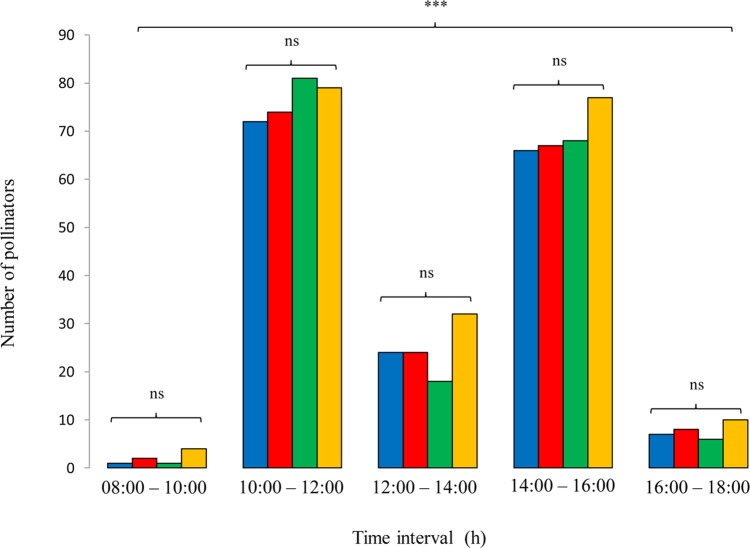
Number of pollinators in four populations (blue scale bars, Cassano; red scale bars, Corato; green scale bars, Acquarola; yellow scale bars, Lucignano) of *I. tuberosa* divided into time intervals (ns: not significant; ***P < 0.001).

**Figure 3.**
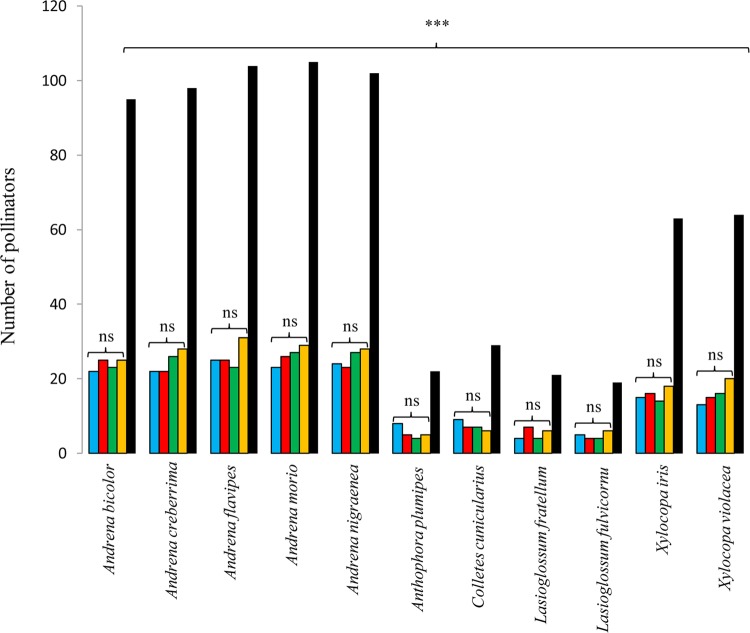
Pollinator species in four populations (blue scale bars, Cassano; red scale bars, Corato; green scale bars, Acquarola; yellow scale bars = Lucignano) and total (black scale bars) of *I. tuberosa* (ns: not significant; ***P < 0.001).

A total of 722 insects were observed in *I. tuberosa* flowers. All the insects collected on *I. tuberosa* flowers were found mainly between 1000 and 1200 h and between 1400 and 1600 h on sunny days (Fig. 2). Nine species were recognized as effective pollinators belonging to five genera of Hymenoptera (*Andrena*, *Anthophora, Colletes*, *Lasioglossum* and *Xylocopa*). Hymenopteran bees are considered the principal pollinating agents of *Iris* species. Consistent with this, *I. tuberosa* were exclusively pollinated by hymenopteran species. Among them, *Andrena* was the dominant genus representing >65 % (504/722) of *I. tuberosa* pollinators (Fig. 3). In particular, the main pollinators were *Andrena nigroaenea*, *A. flavipes*, *A. bicolor*, *A. creberrima* and *A. morio* (Fig. 3). Of the 622 insects (78 %) 565 were males. There were no significant differences between the populations with regard to the number of pollinators (*F*_1,3_ = 1.37, *P* = 0.15 by ANOVA). No pollinator was ever observed moving from one unit to another unit within the same flower. Indeed, the most frequent behaviour for all bees was to visit only one floral unit of a flower and then move to another flower. In all sites, the frequencies of pollinators were not significantly different between ‘tall’ and ‘short’ plants (*F*_1,3_ = 0.81, *P* = 0.38 by ANOVA).

### Natural fruit set and breeding system

**Figure 4.**
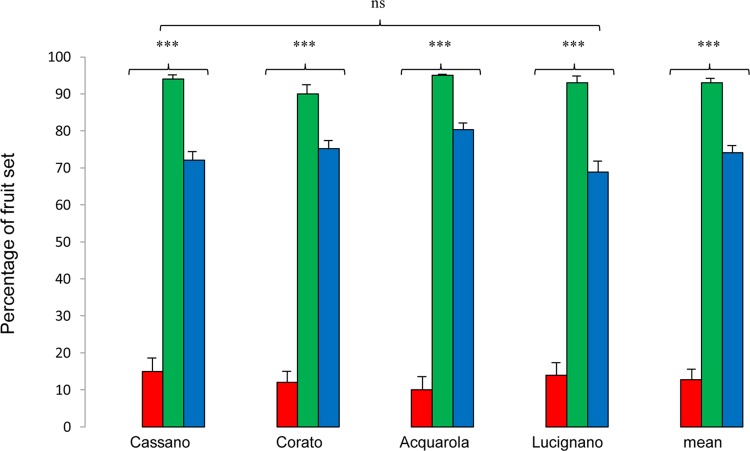
Fruit set in four populations of *I. tuberosa* and mean value for hand-pollination treatments: artificial selfing (red scale bars), outcrossing (green scale bars) and natural open-pollinated (blue scale bars). Spontaneous autogamy and agamospermy are not represented in the figure because the fruit set was 0 % in all populations (ns: not significant; ***P < 0.001).

The natural levels of fruit set in open-pollinated populations ranged from 68 % in Lucignano to 80 % in Acquarola (Table 1). There was no significant difference in fruit set among populations (*F*_3,37_ = 0.69, *P* = 0.33 by ANOVA). Moreover, in each site, fruit set was independent of plant height (*χ*^2^ = 0.16, d.f. = 1, *P* = 0.68). In all populations, fruit set was significantly higher in hand-pollinated than in open-pollinated flowers (*F*_3,37_ = 6.67, *P* < 0.001 by ANOVA). Fruit set by agamospermy and spontaneous autogamy was 0 % in all sites. From the flowers bagged and self-pollinated, low numbers of fruit (mean 13.29 %) were produced (Fig. 4). Pollination treatments of intrapopulation xenogamy gave 90–95 % of the fruit set (Fig. 4). All examined populations of *I. tuberosa* showed low SCI values as well as significantly lower fruit sets after hand selfing than after outcrossing (Table 2). In all the examined populations, the PLI was high, ranging from 0.18 (Corato) to 0.26 (Lucignolo) (Table 2).

**Table 2.** Fruit set after hand selfing and hand outcrossing, SCI and PLI of four *I. tuberosa* populations.

	Fruit set (±SE)			
	Hand crossing	Hand selfing	SCI	PLI
Cassano (*n* = 12)	94.65 ± 2.10	15.22 ± 0.95	0.16	0.24
Corato (*n* = 12)	90.96 ± 1.85	12.54 ± 0.93	0.13	0.18
Acquarola (*n* = 12)	95.18 ± 2.21	10.77 ± 0.92	0.11	0.19
Lucignano (*n* = 12)	93.71 ± 1.95	14.62 ± 0.98	0.15	0.26
Mean	93.63 ± 2.02	13.29 ± 0.95	0.14	0.21

## Discussion

This study showed that *I. tuberosa* were pollinated exclusively by hymenopteran species, mainly during sunny days, and that plant and floral size did not affect fruit set and pollinator attraction.

Field observations revealed that hymenopteran insects belonging to *Andrena*, *Anthophora*, *Colletes*, *Lasioglossum* and *Xylocopa* were common pollinators of *I. tuberosa.* During morning observations on rainy or windy days, I did not observe any insects within flowers, excluding pollination by sheltering as suggested in *Iris atropurpurea* (Watts *et al.* 2013). These data are consistent with previous studies, indeed *Xylocopa violacea* was identified as a pollinator of *I. tuberosa* (Arcangeli 1895), and small (*Apis* sp.) and large (*Bombus* sp. and *Xylocopa* sp.) apoids, such as solitary bees (*Anthophora* sp. and *Eucera* sp.), were the most common pollinators of other Mediterranean *Iris* species (Sapir *et al.* 2006; Segal *et al.* 2006; Imbert *et al.* 2014).

Hand pollinations and field observations indicated that capsules were produced only from open or hand, cross-pollinated flowers, while fruit set did not occur by apomictic processes, in which stigmas of emasculated flowers were covered to avoid pollen contamination. Moreover, the low number of capsules produced by hand self-pollination, as well as low values of self-compatibility index, suggests that *I. tuberosa* is rather self-incompatible. Other Mediterranean *Iris* species are largely self-incompatible (Arafeh *et al.* 2002; Sapir *et al.* 2006; Imbert *et al.* 2014), while selfing rates in other *Iris* species range from 21.4 to 74.1 % (Wilson 2001; Wheelwright *et al.* 2006; Imbert *et al.* 2014). In *I. tuberosa*, self-incompatibility is expressed within the ovary where ovarian grooves have enlarged epidermal cells that produce a floccular secretion that evidently provides discriminatory activity to incoming pollen tubes. In some cases, an indication of post- as well as prezygotic self-incompatibility is expressed within the ovule where embryos abort early in their development (Grilli Caiola and Brandizzi 1997).

The lower values of natural fruit set compared with supplementary artificial pollination treatments in all populations, the low selfing rates and the high values of PLI emphasize the dependence of *I. tuberosa* on insect pollination for its reproductive success. Hand pollination produced more fruits than naturally pollinated plants, and thus, sexual reproduction in *I. tuberosa* is essentially limited by pollinators, not by energy resource availability. This was unexpected, since this plant species is pollinated by many different species of insects, while it is widely assumed that specialized plants will be more prone to experiencing pollen limitation than generalized plants (Knight *et al.* 2005). These results contradict other studies (Ehlers 1999; Červenková and Münzbergová 2013) but, on the contrary, are in agreement with many studies using similar experimental methods to detect pollen limitation in Rosaceae and Brassicaceae (Pflugshaupt *et al.* 2002; Sandring and Agren 2009). Moreover, these results confirm that pollen limitation is more common in self-incompatible species than in species that are self-compatible (Mustajarvi *et al.* 2001). Wheelwright *et al.* (2006) found pollinator limitation to be common in *Iris versicolor* in which fruit set was increased compared with open-pollinated controls. I was also unable to assess pollinator limitation over the course of a plant's entire lifetime. Some species may appear pollinator limited in one reproductive season while effectively being resource limited over the course of several seasons (Ackerman and Montalvo 1990).

Numerous studies have shown that an increase in plant size and floral size makes the plant more attractive, increasing the frequency of pollinator visitations (Sahli and Conner 2007; Frey and Bukoski 2014; Yamada and Masayuki 2014) and leading to an increase in fruit set (Johnson *et al.* 1995; Kawarasaki and Hori 1999). In many plant species, there is a positive correlation between the degree of pollinator attractiveness and the display size of plants (Klinkhamer and De Jong 1990; Kawarasaki and Hori 1999; Sahli and Conner 2007). Contrary to this expectation, I found no evidence for pollinator-mediated selection based on plant and floral size. Indeed, little difference in the number of captured insects was found between ‘tall’ and ‘short’ plants, and I found that fruit set was not correlated with plants having different display sizes. These findings suggested that display size did not seem to influence pollination success of *I. tuberosa*. Similar results were shown for *Iris haynei*, while in populations of *I. atropurpurea* and *I. gracilipes* the selection noted on floral size suggested an advantage for larger flowers and taller plants in attracting pollinators (Ishii and Sakai 2001; Lavi and Sapir 2015). In particular Ishii and Sakai (2001) showed that the number of pollinator visits per plant increased with display size, while the number of pollinator visits per flower did not increase. Their findings suggest that a larger display size promotes successive visits within the plant, but does not increase pollinator visits to individual flowers. Moreover, this relationship between plant size and pollinator visitations was not constant across years and populations (Lavi and Sapir 2015), suggesting that different factors may obscure this selection. For example, Robertson and Macnair (1995) suggested that when plant density is relatively high, pollinators visit flowers on the plants in the population at an equal rate, irrespective of display size. In addition, the extreme floral size of *I. atropurpurea* (floral diameter >64 mm and floral length >78 mm) relative to the smaller floral size of *I. tuberosa* could explain the presence of pollinator-mediated selection only in *I. atropurpurea.* Plants such as *I. atropurpurea* with larger floral displays and growing in patches contain up to hundreds of flowers that attract pollinators from greater distances, while the presence of a single flower and isolated plants (*I. tuberosa*) discourage pollinator visitation, limiting any increase in female reproductive success that would otherwise occur in a multi-flowered species (Jersáková *et al.* 2006).

## Conclusion

This study quantifies the role of pollinators on reproductive success in *I. tuberosa*. Pollinators visited tall/short plants and large/small flowers in equal proportion, thus suggesting that plant and floral display size do not affect pollinator behaviour and reproductive success in this species. I hypothesize that additional selection factors can act on floral traits to explain the lack of pollinator-mediated selection based on floral size. Further studies are needed to verify the relative importance of pollinator limitation. For example, the surrounding vegetation context (i.e. the presence of rewarding co-flowering species which could have either negatively, through competition for pollinators, or positively by means of a magnet species effect and floral mimicry, Pellegrino *et al.* 2008) or different pollination strategies (i.e. the scent production or night-sheltering that can help to attract pollinators) are important, relatively understudied components that should be considered to understand the evolution and functional significance of floral traits.

## Sources of Funding

This research was made possible, in part, through a grant from the Italian Ministry of Education, University and Research (ex MURST 60 %).

## Conflicts of Interest Statement

None declared.
